# Wear behavior at margins of direct composite with CAD/CAM composite and enamel

**DOI:** 10.1007/s00784-023-04883-w

**Published:** 2023-02-06

**Authors:** Lippo Lassila, Rudolf Novotny, Eija Säilynoja, Pekka K. Vallittu, Sufyan Garoushi

**Affiliations:** 1 Department of Biomaterials Science and Turku Clinical Biomaterial Center-TCBC, Institute of Dentistry, University of Turku, Turku, Finland; 2 Research Development and Production Department, Stick Tech Ltd—Member of GC Group, Turku, Finland; 3 City of Turku Welfare Division, Oral Health Care, Turku, Finland

**Keywords:** Two-body wear, Dental composite, Fiber-reinforced composite, Marginal breakdown

## Abstract

**Objectives:**

The aim was to investigate the two-body wear at the marginal area between direct filling composites and substrate of CAD/CAM composites or enamel.

**Materials and methods:**

Flat specimens were prepared from CAD/CAM composites (CERASMART 270 and SFRC CAD) and bovine enamel. A box-shaped cavity cut into CAD/CAM composites and enamel surfaces was made. The prepared cavity in CAD/CAM composites was treated with a primer, while in enamel, the cavity was treated with an adhesive. Three conventional composites (Universal Injectable, G-aenial A’Chord, and Filtek Bulk Fill) and one short fiber composite (everX Flow) were placed and cured in the prepared cavities. A two-body wear test was conducted with 15,000 chewing cycles using a dual-axis chewing simulator. The specimens (*n* = 5/per group) were positioned to produce wear (load = 20 N) across the marginal area between filling composites and substrates. The wear depth was analyzed using a 3D optical profilometer. SEM was used to evaluate the wear behavior and margins between the filling and substrate materials.

**Results:**

All composites used displayed different wear behavior (20–39 µm) (*p* < 0.05). The highest wear values were recorded for A’Chord and Filtek, while the lowest values were for Injectable and CERASMART 270. The data analysis showed that the wear behavior of substrate materials depends on the filling materials used at margins (*p* < 0.05). The marginal breakdown was seen only between bovine enamel and filling composites.

**Conclusions:**

The use of the two-body wear simulation method revealed important information about the behavior of the filling composites at the marginal area with CAD/CAM composites or bovine enamel substrates.

**Clinical relevance:**

The marginal breakdown related to the material combination at the bonding region.

## Introduction

Large cavities in posterior teeth can be restored using indirect composites instead of direct filling composites because indirect materials allow for better control over proximal contacts, anatomic form, esthetics, and polymerization shrinkage [1]. Computer-aided design and computer-aided manufacturing (CAD/CAM) composites could also provide other advantages like improved mechanical properties, adequate wear resistance, less discoloration, and less residual monomer [2, 3]. Additionally, compared to ceramic materials, these polymer-based composites are less likely to chip when cut to extremely thin dimensions, are not heated, are less abrasive to the opposing enamel, and are easier to repair in case of failure [4, 5]. These characteristics make CAD/CAM composite restorations suitable for clinical use; however, some concerns exist regarding their long-term clinical performance, as some of their mechanical properties could be lower than ceramics [6, 7]. Recently, an experimental short fiber–reinforced CAD/CAM composite was introduced with the aim of improving the fracture toughness of conventional particulate-filled CAD/CAM composites [8–11]. Studies demonstrated promising performance when mechanical, optical, surface, and bonding properties were tested.

In general, failures of CAD/CAM composite restorations may be a result of secondary caries or chipping/fracture or wear [5]. The direct repair of failed restorations using filling composite is preferable since it is more conservative, less expensive, and less time-consuming; minimizes tooth tissue loss; and reduces pulpal trauma. However, the repair of defective CAD/CAM restorations may be a challenging clinical situation. One of the primary challenges is the marginal breakdown between the two materials. During mastication, restorations are subjected to repeated mechanical forces, and as a result, wear occurs. Occlusal wear causes loss of the anatomical shape and marginal breakdown of the composite restorations and the repair composite [12]. Therefore, wear resistance of composite restorations is important for long-term success [13, 14]. The most recent research on CAD/CAM composite repair is limited and mainly focused on evaluating repair bond strengths to find the best repair protocols [5, 8]. On the other hand, many studies in existing literature have evaluated the wear of different direct and CAD/CAM composites [14–16]. Results from these investigations on the two-body wear behavior show that the wear damage pattern of composites is closely related to their microstructure. However, none of these studies investigated the wear at the marginal area between both CAD/CAM and repair composites. Thus, the aim of this study was to investigate the two-body wear at the marginal area between repair composites and CAD/CAM composites or enamel. The null hypothesis tested was that there was no significant difference regarding the mean wear depth values between the materials at the marginal area.

## Materials and methods

Four commercially available filling composites (Table 1), one flowable particulate-filled (G-aenial Universal Injectable), two packable particulate-filled (G-aenial A’Chord and Filtek Bulk Fill), and one flowable short fiber–reinforced (everX Flow) were used in this study as repair composites. Two CAD/CAM composites were used as substrate material (Table 1); they consisted of one experimental short fiber–reinforced (SFRC CAD) and one particulate-filled (CERASMART 270).

**Table 1:** Materials used in the study

Material (code)	Manufacturer	Composition
G-aenial Universal Injectable (In)	GC Corp	Dimethacrylate monomers, barium glass, and silica 69 wt%
everX Flow (eX)	GC Corp, Tokyo, Japan	Bis-EMA, TEGDMA, UDMA, short glass fiber (200–300 µm and Ø7 μm), and barium glass 70 wt%
G-aenial A’Chord (Ac)	GC Corp	UDMA, Bis-EMA, Bis-GMA, TEGDMA, Bis-MEPP, prepolymrized silica, and barium glass 81 wt%
Filtek Bulk Fill (Fb)	3 M, St. Paul, MN, USA	AUDMA, UDMA, DDDMA, 76.5 wt% zirconia/silica, and ytterbium trifluoride fillers in nanometer scale (av. Ø 20 nm)
SFRC CAD (FC)	Experimental	UDMA, TEGDMA, short glass fiber (200–300 µm and Ø7 μm), barium glass 77 wt%
CERASMART 270 (CS)	GC Corp	Bis-MEPP, UDMA, dimethacrylate, silica (20 nm), and barium glass (300 nm) 71 wt%
G-Premio BOND (universal bonding agent)	GC Corp	MDP, 4-MET, MEPS, methacrylate monomer, acetone, water, initiator, and silica
G-Multi PRIMER	GC Corp	Ethyl alcohol (90–100%), phosphoric acid ester monomer (1–5%), and dimethacrylate (1–5%)

*Bis-GMA*, bisphenol-A-glycidyl dimethacrylate; *TEGDMA*, triethylene glycol dimethacrylate; *UDMA*, urethane dimethacrylate; *Bis-MEPP*, Bis (p-methacryloxy (ethoxy) 1–2 phenyl)-propane; *Bis-EMA*, ethoxylated bisphenol-A-dimethacrylate; *AUDMA*, aromatic urethane dimethacrylate; *DDDMA*, 12-dodecanediol dimethacrylate; *4-MET*, 4-methacryloyloxyethyl trimellitate; *MDP*, 10-methacryloyloxy-decyl dihydrogen phosphate; *MEPS*, methacryloyloxyalkyl thiophosphate methylmethacrylate; *wt%*, weight percentage

### Specimen preparation

A total of 40 block-shaped specimens (14 mm length × 12 mm width × 3 mm thick) were prepared (*n* = 20/substrate CAD/CAM material) using a low-speed diamond saw (Struers, Glasgow, Scotland). Each specimen was polished flat using silicon carbide papers (#1200 grit) at 300 rpm and under water cooling using an automatic grinding machine (Rotopol-1; Struers) for 30 s. A box-shaped cavity (6 mm length × 2 mm width × 2 mm depth) was prepared on each specimen with a carbide bur under water cooling. Each cavity was treated with a primer (G-Multi PRIMER) according to the manufacturers’ instructions. Then, direct filling composites (*n* = 5) were applied into the drilled cavities in one increment, flattened (mylar strip and glass slide), and light cured (Elipar™ S10, 3 M ESPE, Germany) for 40 s from the top surface in different overlapping sections keeping the light tip in contact to the specimens. The wavelength of the light was between 430 and 480 nm, and the light intensity was 1200 mW/cm^2^ (MARC Resin Calibrator, BlueLight Analytics Inc., Canada). Then, each specimen was polished flat using silicon carbide papers from #1200 to #4000 grit at 300 rpm and under water cooling using an automatic grinding machine (Rotopol-1; Struers) for 30 s per grit.

Bovine incisors had their roots removed using a low-speed diamond saw and were used as control substrate material (*n* = 20). The labial surfaces (bovine enamel) were ground flat on coarse silicon carbide paper. Cavity preparation was made as described with CAD/CAM substrate materials. Each cavity was acid-etched (37% phosphoric acid) and treated with adhesive using a one-bottle universal bonding agent (G-Premio BOND) according to the manufacturers’ instructions. Then, direct filling (repair) composites were applied into the prepared cavities as described before. All specimens were stored for 24 h in water (37 °C) before wear testing.

### Two-body wear

**Fig. 1:**
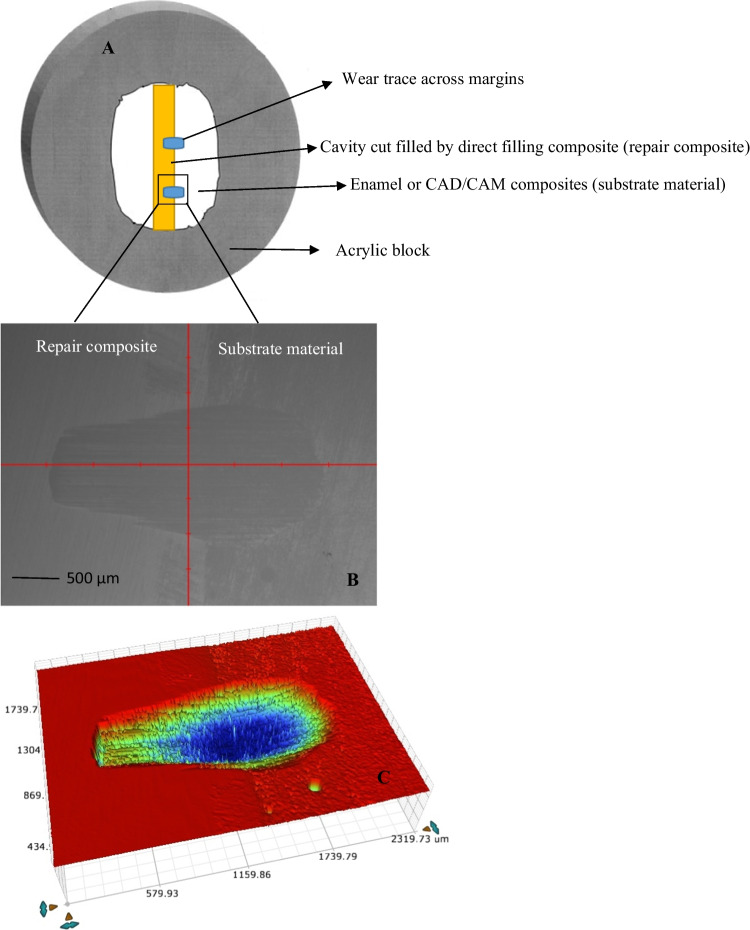
Schematic figure of the tested specimen (**A**) showing the wear trace (blue color) across the margin between filling (yellow color) and substrate (white color) materials. **B** and **C** are typical 2D and 3D surface profiles of the wear trace at the marginal area

Specimens (*n* = 60) were placed in an acrylic resin block to test the localized wear. A two-body wear test was accomplished using a chewing simulator (CS-4.2, SD Mechatronik, Germany) that has two chambers simulating horizontal and vertical movements concurrently with water. Each chamber was composed of an upper sample holder that fastened the loading tip with a screw and a lower sample holder made of plastic where the composite/enamel specimen was embedded. The specimens were embedded in the lower sample holder in acrylic resin to be used as antagonistic wear material. The manufacturer’s standard loading balls (Steatite ball, Ø 6 mm) were embedded in acrylic resin in the upper sample holders, and a fastening screw was used to fix them. The loading ball was positioned close to the marginal area between repair composite and substrate material (CAD/CAM composite or enamel) in order to produce a sliding wear trace across margins (*n* = 2/specimen) (Fig. 1). A total of 15,000 simulation chewing cycles (vertical movement 3 mm and lateral movement 2 mm) were performed at 1.5 Hz with a vertical weight of 2 kg, simulating a chewing force of 20 N. Wear patterns (*n* = 10/group) were scanned with a 3D optical profilometer (Bruker Nano GmbH, Model no. Contour GT-K, Berlin, Germany) and then analyzed with Vision64 Map software for material loss measurements. Total wear depth values (µm) were obtained from various sites, according to the average of the deepest points of all profile scans.

Additionally, after evaluating the vertical wear depth, representative specimens of each material were examined by scanning electron microscopy (SEM, LEO, Oberkochen, Germany) to analyze the wear facets and margins. Before SEM examination, the specimens were gold sputtered with a gold layer in a vacuum evaporator using a sputter coater (BAL-TEC SCD 050 Sputter Coater, Balzers, Liechtenstein).

### Statistical analysis

The data were statistically analyzed with SPSS version 23 (SPSS, IBM Corp.) using a two-way analysis of variance (ANOVA) at the *p* < 0.05 significance level, followed by a Tukey HSD post hoc test to determine the differences between the groups. Wear depth values were the dependent variables, while the type of substrate material and direct filling composites were the independent variables.

## Results

**Fig. 2:**
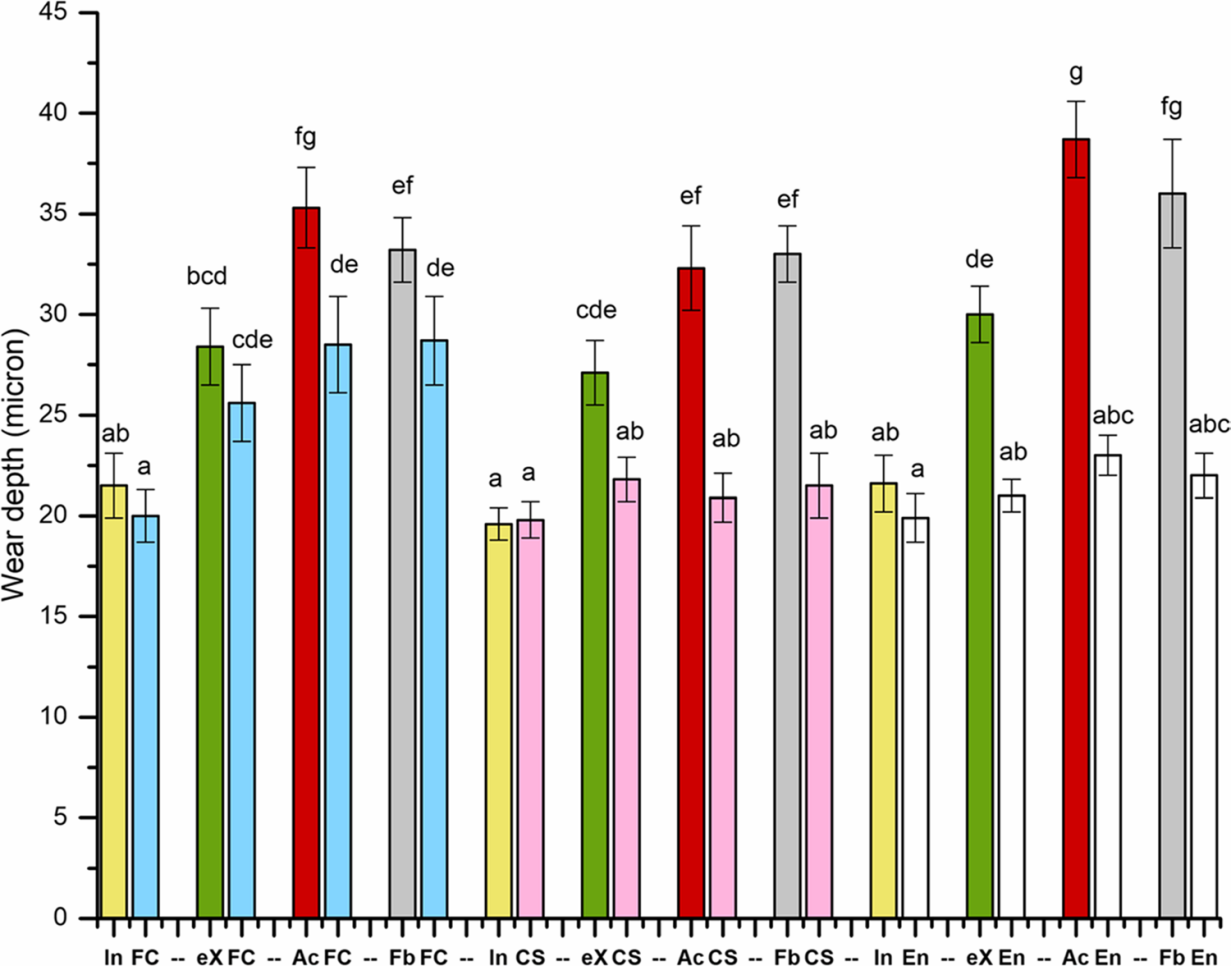
The average of maximum wear depths at the marginal area between filling and substrate materials. En refers to enamel (control). The same letters above the bars represent non-statistically significant differences (*p* > 0.05) among the materials

The result of wear depth at the marginal area between direct filling and substrate materials is presented in Fig. 2. In general, the wear resistance of substrate materials (indirect CAD/CAM composite or bovine enamel) was better than direct filling composites except for G-aenial Universal Injectable which had comparable wear depth values to CAD/CAM composites and bovine enamel (*p* > 0.05).

The data analysis showed that the wear behavior of substrate materials depended on the filling composites used at the marginal area (Fig. 2). The experimental SFRC CAD/CAM composite exhibited higher wear depth values in comparison with other substrate materials (*p* < 0.05) when packable highly filled composites (G-aenial A’Chord and Filtek Bulk Fill) are used at the marginal area. However, there was no significant difference (*p* > 0.05) when G-aenial Universal Injectable was used (Fig. 2). CERASMART 270 showed similar wear behavior to that of bovine enamel (*p* > 0.05).

There was a statistically significant difference among the investigated direct filling composites in the wear depth values (*p* < 0.05). The lowest average wear depth was found for G-aenial Universal Injectable, while G-aenial A’Chord and Filtek Bulk Fill presented the highest average wear depth values (*p* < 0.05).

**Fig. 3:**
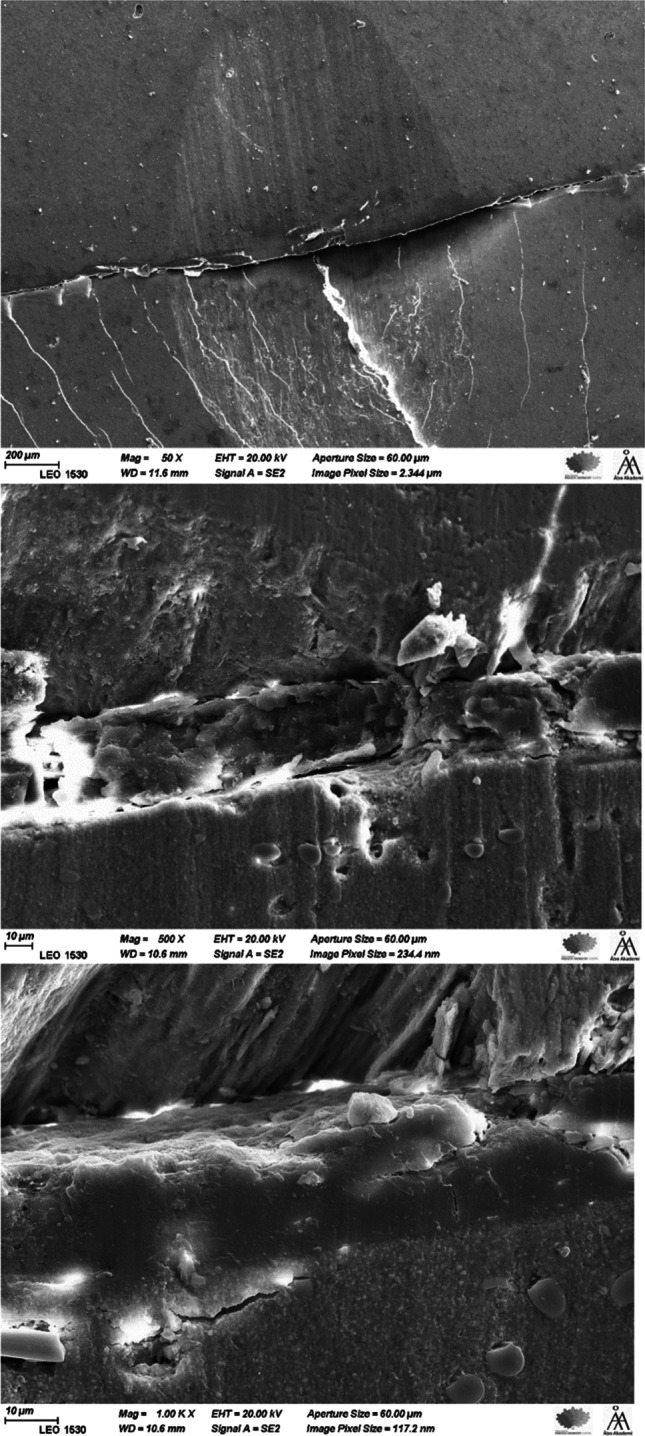
SEM images (50, 500, and 1000 ×) showing the breakdown of the margins between bovine enamel and filling composite (eX) after the wear test

**Fig. 4:**
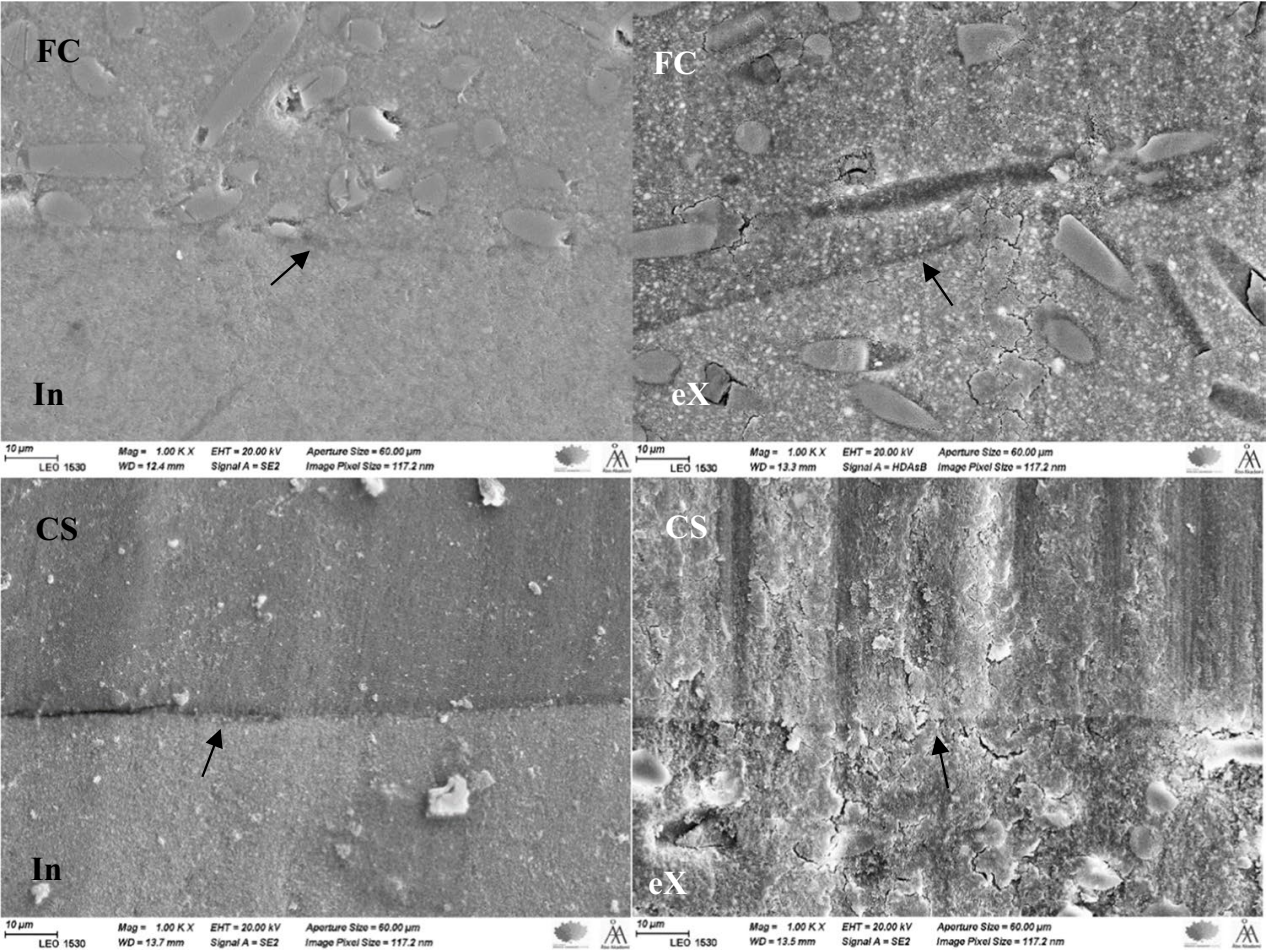
SEM images (1000 ×) showing the margins (arrows) between filling and substrate (CAD/CAM) composites after the wear test

Figure 3 shows the marginal breakdown between direct filling composite (everX Flow) and bovine enamel. The trend was similar for all filling composites. On the other hand, direct filling composites maintain good margins with CAD/CAM composites despite the difference in wear depth values (Fig. 4).

## Discussion

Wear behavior of dental composites especially at the marginal area is important as it can affect their clinical service and occlusal contacts overtime. In this study, a two-body wear (impact-sliding wear) test was selected because it is practical and commonly used [14, 16–18]. The used chewing simulation set-up included the vertical application of masticatory load to simulate direct contact between the test specimen and its antagonist, as well as additional lateral movement of the specimen carrier. Therefore, both abrasive and fatigue wear were simulated in one chewing simulation assay. Within the limitations of the present study, the null hypothesis that there was no significant difference in wear resistance at the marginal area between the tested repair and substrate composites was rejected.

When comparing direct filling composites to CAD/CAM composites, most studies reported generally higher wear depth values for direct filling composites than CAD/CAM composites [10, 14, 19, 20]. In accordance, the tested packable highly filled composites (G-aenial A’Chord and Filtek Bulk Fill) showed significantly higher wear depth values than CAD/CAM composite (CERASMART 270) made partly by similar manufacturer-specific filler/silane technology. This may be explained by the fact that ideal curing conditions of CAD/CAM composite contribute to better polymer cross-linking [10, 14]. With smaller filler sizes and more homogeneous nanofillers, CERASMART 270 exhibited higher wear resistance than packable composites with larger fillers. On the other hand, direct particulate-filled flowable composite (G-aenial Universal Injectable) and CAD/CAM composite (CERASMART 270) with similar manufacturer filler/silane technology showed no significant differences in wear depth values (Fig. 2). Considering that flowable composites have a lower filler weight than CAD/CAM or packable composites, this result may not have been expected. This may partly be due to the elastic deformation of the flowable composite matrix which provides some shock-absorbing ability [20, 21]. However, composite wear is a complicated process, and so far, no clear consensus could be found in the literature [14]. Results in the literature vary: some studies found a positive [22, 23] or a negative [17, 24] correlation between surface hardness and wear resistance, while others did not find an interdependency [25, 26]. Some other studies found that the wear resistance of the composite can be influenced not only by the fillers’ size and volume or inter-particle distance, but also by the composition of the resin matrix and by the silane coupling agent which improves the bond of fillers and resin matrix [26–28]. Furthermore, a number of researchers have hypothesized that fracture toughness, elastic modulus, or flexural strength could all be indicators of clinical wear [12, 29].

In this study, SFRCs (SFRC CAD and everX Flow) revealed comparable wear depth values compared to some evaluated commercial particulate-filled composites (Fig. 2). Thereby, microfiber filler loading was not deteriorating the wear of the SFRCs. This is in accordance with previous studies that showed good wear resistance behavior of flowable SFRC in comparison with different particulate-filled composites [10, 30]. These results give an indication that short fiber–reinforced composites (SFRC CAD and everX Flow) could be used safely in a wider range of clinical applications. The variation in the wear depth values between SFRC CAD and the flowable SFRC composite (everX Flow), despite having a similar glass fiber content, was probably due to differences in the amount of the particulate filler content. The glass barium particles are abundantly available in the experimental SFRC CAD/CAM (77 wt%) compared to the flowable SFRC (70 wt%). Furthermore, SFRC CAD is subjected to heat curing under high temperature and pressure. These optimum curing conditions can participate in improving the wear resistance [10].

It was interesting to note that the wear depth values of bovine enamel did not significantly differ from those of G-aenial Universal Injectable and CERASMART 270 (Fig. 2). This is in accordance with Leinfelder [31], who assumed that hybrid ceramics exhibit a wear close to natural teeth and show similar deformation capacity. However, it is important to realize that there are structural and mechanical differences between the human enamel and the bovine enamel used in this study which make it less wear resistant [32, 33].

In this study, marginal breakdown results were in agreement with a previous study by Ferracane and Condon, which reported enamel degradation at the margin with direct filling composites [12]. The enamel failure seems to occur by delamination of the enamel rods from one another, as evidenced by the exposure of the rod surfaces in the electron micrographs (Fig. 3). It is likely that the cyclic load at the margins first produces a wear facet in the composite, leading to a loss of material at the marginal area, and enamel was left unsupported. Consequently, the enamel began to chip under the contact load. This kind of failure (brittle fracture) seems to be caused by differences in the elastic modulus of materials at the marginal area. Interestingly, all tested filling composites maintain good margins with CAD/CAM composites despite the difference in wear depth values (Fig. 4). The resin matrix, with a relatively lower elastic modulus, exhibited plastic deformation when subjected to contact load (impact), counteracting part of the stress. However, it is logical to hypothesize that this phenomenon may also be related to some other physical properties of the composites, specifically fracture toughness differences between enamel and composite material [12, 13].

Recent studies, using a two-body or impact-sliding wear test set-up, claimed that as the wear test progressed, wear debris was generated when the antagonist slid from the impact to the sliding part, contributing to the three-body wear [16, 34, 35]. However, this issue needs to be further investigated.

Another important aspect is the adhesive system used at the marginal area that might be the potentially weak link in the failure mechanism. It has previously been suggested that marginal integrity may be enhanced when composites are tightly bonded to the cavity margin [36, 37]. G-Premio BOND is a single-stage adhesive that contains both acidic hydrophilic primer and hydrophobic resin in one bottle, but it has no fillers. Although not all universal adhesive systems contain nanofiller particles, the addition of nanofillers increases the mechanical properties and wear resistance of the adhesive material itself [38].

Because of the optimum curing condition of CAD/CAM composites, a number of techniques have been suggested to improve the bond strength of composite repair through roughening or etching the substrate surface with hydrofluoric acid gel or air-particle abrasion or using adhesion primers [39, 40]. Application of G-Multi PRIMER after cavity preparation in our investigation appears to be helpful in preserving the margins between filling and CAD/CAM composites (Fig. 4). According to Khan and his colleagues, the primary role of the universal primer is to dissolve the surface of substrate composite and improve the penetration of monomers of repair resin composite into the substrate composite, which forms solid adhesive interface bonding after curing [41, 42]. Furthermore, the irregular surface structure created by grinding or a rotary instrument may also induce micromechanical interlocking between substrate and repair composites [40].

The results of this investigation must be seen in the perspective of some limitations. Only two-body wear of flat composite specimens against one artificial antagonist (Steatite ball) was evaluated. Different results might have been obtained with a three-body wear test or anatomical-shaped specimens or with other antagonists, such as human enamel.

## Conclusion

The use of the two-body wear simulation method revealed important information about the behavior of the direct filling composites at the marginal area with CAD/CAD composites or bovine enamel substrates. For CAD/CAM composite repair, the use of flowable composite is a good option in terms of wear behavior.

## Data Availability

The data presented in this study are available on reasonable request from the corresponding author.
